# Comparative multiphase CT radiomics for differentiating isolated hepatic metastasis from intrahepatic cholangiocarcinoma: a two-center study of native, subtraction, peritumoral, and fusion models

**DOI:** 10.3389/fonc.2026.1907000

**Published:** 2026-07-30

**Authors:** Yanyan Zhou, Qianlian Wu, Hejia Zhang, Peng Chen, Chengmeng Zhang, Jian Shen, Junmei Wang, Chuanxian Liu, Zhimin Ding, Liangshan Li

**Affiliations:** 1 Department of Radiology, Changxing Hospital of Traditional Chinese Medicine, Huzhou, China; 2 Department of Radiology, Yijishan Hospital Affiliated to Wannan Medical University, Wuhu, China; 3 School of Medicine, Linyi University, Linyi, China; 4 Department of Radiology, Huzhou Central Hospital, Huzhou, China; 5 Department of Radiology, Jiaxing Hospital of Traditional Chinese Medicine Affiliated to Zhejiang Chinese Medical University, Jiaxing, China

**Keywords:** computed tomography, external validation, intrahepatic cholangiocarcinoma, liver metastasis, radiomics, subtraction imaging, XGBoost

## Abstract

**Background:**

Isolated hepatic metastasis (IHM) and intrahepatic cholangiocarcinoma (IMCC) may show overlapping appearances on multiphasic contrast-enhanced computed tomography (CT). A quantitative imaging approach may help improve noninvasive differentiation. This study aims to develop and externally validate multiphasic contrast-enhanced CT radiomics models for differentiating IHM from IMCC and to evaluate the diagnostic value of native, subtraction, peritumoral, and fusion information.

**Methods:**

This retrospective two-center study included patients with pathologically confirmed IHM or IMCC who underwent multiphasic liver CT at Huzhou Central Hospital or Yijishan Hospital Affiliated to Wannan Medical University between January 2023 and December 2025. The final cohort included 227 patients. Center 1 was used as the training cohort (n=115; IHM, n=68; IMCC, n=47), and center 2 was used as the external validation cohort (n=112; IHM, n=60; IMCC, n=52). Clinical baseline variables included sex, age, and serum tumor markers. Radiomics features were extracted using PyRadiomics from unenhanced CT images (C), arterial phase CT images (A), venous phase CT images (V), and subtraction images M=A-C, N=V-C, and P=A-V. Ten prespecified models were constructed to represent native-image, subtraction-image, peritumoral, and fusion information levels. Feature selection was performed in the training cohort using median imputation, variance filtering, and ANOVA F-test-based SelectKBest. XGBoost classifiers were tuned using five-fold stratified cross-validation in the training cohort and evaluated once in the external validation cohort.

**Results:**

In the training cohort, apparent AUCs ranged from 0.864 to 0.978. The subtraction-total model based on M+N+P intratumoral features showed the highest numerical external validation performance, with an AUC of 0.835 (0.755-0.907), accuracy of 0.759, sensitivity of 0.800, and specificity of 0.712. The intra-plus-peritumoral model achieved an external validation AUC of 0.800 (0.713-0.882). The plain unenhanced model achieved an AUC of 0.821 (0.739-0.900), whereas the native C+A+V model and ultimate fusion model achieved AUCs of 0.809 (0.725-0.888) and 0.800 (0.714-0.884), respectively. Pairwise DeLong tests among XGBoost models did not show statistically significant differences after multiple-comparison correction.

**Conclusion:**

Multiphase contrast-enhanced CT radiomics can differentiate IHM from IMCC. Subtraction-based intratumoral models demonstrated the highest numerical external validation performance, although the advantage was not statistically significant after multiple-comparison correction. These findings indicate the potential discriminatory value of enhancement-difference information.

## Introduction

Intrahepatic cholangiocarcinoma and hepatic metastasis are common malignant liver lesions with different treatment strategies and prognostic implications (1–3). In clinical practice, isolated hepatic metastasis and intrahepatic cholangiocarcinoma present as solitary liver masses with overlapping enhancement patterns, making preoperative differentiation challenging (4). Accurate noninvasive discrimination may support surgical planning, systemic therapy decisions, and multidisciplinary management.

Multiphase liver CT provides morphologic and dynamic contrast-enhancement information (4–6). Native CT images reflect tumor morphology and baseline attenuation heterogeneity, whereas contrast-enhanced and subtraction images may better characterize enhancement kinetics (7, 8). Peritumoral regions may also contain information related to tumor invasion, local liver reaction, and microenvironmental alterations (9, 10). However, whether these different information layers provide incremental diagnostic value remains uncertain.

Radiomics enables high-throughput extraction of quantitative imaging features from routinely acquired medical images, including intensity, shape, and texture descriptors that may function as noninvasive imaging biomarkers (11–15). For clinical translation, radiomics studies require standardized feature extraction, reproducible preprocessing, transparent feature selection, independent validation, and clinically interpretable evaluation (13–16). Despite its promise, radiomics remains limited by scanner heterogeneity, segmentation variability, feature instability, and incomplete external validation, which should be explicitly considered when developing predictive models (13–16). This study developed ten prespecified CT radiomics models and externally validated them across two centers. The main objective was to evaluate whether native CT, subtraction imaging, peritumoral information, and fusion information differ in their ability to distinguish IHM from IMCC.

## Methods

### Patients

**Figure 1:**
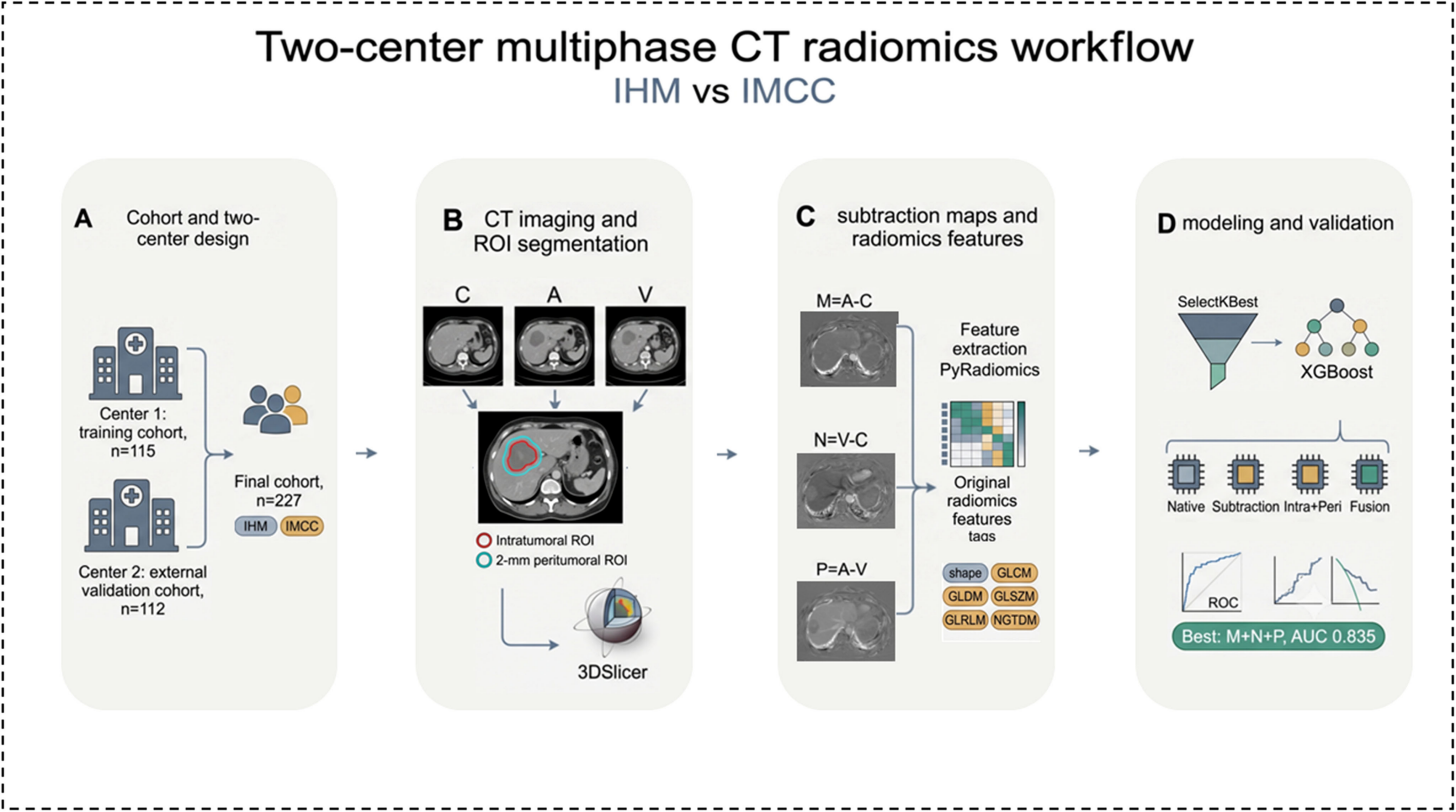
Patient inclusion and modeling workflow.

This retrospective diagnostic modeling study included patients from two centers: Huzhou Central Hospital (center 1) and Yijishan Hospital Affiliated to Wannan Medical University (center 2). Center 1 was used as the training cohort and center 2 as the external validation cohort. Consecutive patients between January 2023 and December 2025 were screened. Candidate patients had pathologically confirmed IHM or IMCC and available multiphasic liver CT. The final study cohort included 227 patients. This study was approved by the institutional ethics committee (approval number: 2026-86), and the requirement for informed consent was waived because of the retrospective design. The overall cohort assembly and modeling workflow are summarized in **Figure 1**.

The inclusion criteria were (1): patients with solitary hepatic lesions histopathologically confirmed asIHM or IMCC via surgical resection or needle biopsy; (2) patients who underwent complete preoperative multiphasic contrast-enhanced liver CT examinations (including unenhanced, arterial, and venous phases) at our institution or participating sub-centers. The exclusion criteria were: (1) patients who received any tumor-related treatments prior to the CT examination, such as transcatheter arterial chemoembolization (TACE), ablation, or systemic antitumor therapies; (2) images with severe motion artifacts, metallic artifacts, or sub-optimal phase timing that hindered accurate lesion boundary delineation or radiomics feature extraction; (3) patients with multiple hepatic metastases or concurrent primary malignancies at other sites.

### CT acquisition and image sets

At center 1 (Huzhou Central Hospital), CT examinations were performed using a 96-row dual-source CT scanner (SOMATOM Force, Siemens, Germany) or a 320-row spiral CT scanner (Aquilion ONE TSX-301C, Toshiba, Japan). At center 2 (Yijishan Hospital Affiliated to Wannan Medical University), CT examinations were performed using a 128-row dual-source CT scanner (SOMATOM Definition Flash, Siemens, Germany) or a 64-row spiral CT scanner (Brilliance 64-row spiral, Philips, Netherlands).

All contrast-enhanced CT scans were performed using an empiric timing protocol. Patients were placed in the supine position, and the scan range extended from the bilateral diaphragmatic domes to the inferior margin of the pubic symphysis. The acquisition parameters were as follows: tube voltage, 120 kV; tube current, automatic tube current modulation; slice thickness, 5.0 mm; and interslice interval, 5.0 mm. After unenhanced scanning, a nonionic iodinated contrast agent was injected through an antecubital vein at a rate of 2.5 mL/s. At center 1, iopromide (iodine concentration, 300 mg/mL) was used. At center 2, iodixanol (iodine concentration, 350 mg/mL) was used. Arterial phase scanning was initiated 35 s after contrast injection, and venous phase scanning was initiated 90 s after contrast injection. The native CT phases included unenhanced images (C), arterial phase images (A), and venous phase images (V). To minimize misregistration artifacts caused by respiratory motion, a rigid voxel-based image registration was performed to spatially align the multiphasic CT scans prior to subtraction. After registration, all subtraction images were visually checked to identify obvious registration errors or subtraction artifacts. No additional cases were excluded solely because of poor subtraction-image quality beyond the prespecified image-quality exclusion criteria. Three subtraction image sets were analyzed: M=A-C, N=V-C, and P=A-V. M and N were intended to represent net arterial and venous enhancement, whereas P was intended to capture washout or persistent enhancement-related differences.

### ROI segmentation

ROI segmentation was performed using 3D Slicer software (version 5.10.0). The whole tumor was manually delineated slice by slice, and necrotic components within the lesion were included. Two attending radiologists specializing in abdominal imaging, each with more than 5 years of experience, independently performed ROI delineation. A senior radiologist with more than 10 years of experience reviewed the segmentations. Reproducibility analysis showed good agreement, with an interobserver ICC of 0.82 (95% CI: 0.80-0.85) and an intraobserver ICC of 0.84 (95% CI: 0.82-0.85).

### Radiomics feature extraction

Radiomics features were extracted with standardized preprocessing settings consistent with IBSI recommendations where applicable (16), using PyRadiomics (17) and a unified parameter file. Only original features were extracted; no wavelet, Laplacian-of-Gaussian, or other filtered-image features were used. Extracted feature classes included shape, first-order intensity, gray-level co-occurrence matrix, gray-level run-length matrix, gray-level size-zone matrix, gray-level dependence matrix, and neighboring gray-tone difference matrix features. Preprocessing included resampling to 1 x 1 x 1 mm3 spacing, a gray-level bin width of 25 HU, B-spline interpolation for images, and image normalization. Feature extraction was performed before model development and was identical for the training and external validation cohorts.

### Model construction

Ten prespecified radiomics models were constructed:

1. Model 1a: C intratumoral features.
2. Model 1b: A intratumoral features.
3. Model 1c: V intratumoral features.
4. Model 1-All: C+A+V intratumoral features.
5. Model 2a: M=A-C intratumoral features.
6. Model 2b: N=V-C intratumoral features.
7. Model 2c: P=A-V intratumoral features.
8. Model 2-All: M+N+P intratumoral features.
9. Model 3: C+A+V features extracted from intratumoral plus 2-mm peritumoral ROI.
10. Model 4: fusion of native, subtraction, and peritumoral information.

### Feature selection and XGBoost modeling

All feature selection and model tuning steps were performed only in the training cohort. Zero-variance features were removed using VarianceThreshold. Candidate features were then ranked using ANOVA F-test-based SelectKBest, and the number of selected features was tuned as a hyperparameter. Candidate values were 5, 10, 20, 50, and all features when applicable. XGBoost was used as the classifier. Hyperparameters, including tree depth, number of estimators, learning rate, subsampling ratio, column sampling ratio, and L2 regularization, were optimized using five-fold stratified cross-validation in the training cohort. The final selected feature set was obtained after refitting the complete feature-selection pipeline on the full training cohort. Final selected feature numbers and key XGBoost hyperparameters for all ten models are provided in **Supplementary Table 1**. The final model was retrained on the full center 1 training cohort and evaluated in the center 2 external validation cohort.

### Statistical analysis

All statistical analyses were performed using R software (version 4.5.1). Clinical baseline characteristics were compared between the training and external validation cohorts. Continuous variables with missing values were imputed using the overall mean before summary and testing. Age was summarized as mean +/- standard deviation and compared using Welch’s t test. Serum tumor markers were summarized as median with interquartile range and compared using Wilcoxon rank-sum tests. Categorical variables were summarized as number and percentage and compared using chi-square or Fisher’s exact tests, as appropriate.

The primary performance metric was the area under the receiver operating characteristic curve (AUC) in the external validation cohort. Internal model performance was assessed using five-fold stratified cross-validation in the training cohort and apparent performance after refitting on the full training cohort. Secondary metrics included accuracy, sensitivity, specificity, positive predictive value, and F1 score. Bootstrap resampling with 2000 iterations was used to estimate 95% confidence intervals for AUC. Pairwise comparisons between XGBoost models were performed using DeLong tests. Calibration curves and decision curve analysis were used to evaluate probability calibration and potential clinical utility. A two-sided P value <0.05 was considered statistically significant.

## Results

### Patient cohort

The final cohort included 227 patients. All included cases were pathologically confirmed. The training cohort from center 1 included 115 patients, including 68 patients with IHM and 47 with IMCC. The external validation cohort from center 2 included 112 patients, including 60 patients with IHM and 52 with IMCC. Baseline diagnosis distribution, age, sex, CA19-9, CEA and AFP were not significantly different between the training and external validation cohorts. Detailed clinical baseline characteristics are provided in **Table 1**.

**Table 1:** Clinical baseline characteristics of the training and external validation cohorts.

Characteristic	Overall	Training cohort (n=115)	External validation cohort (n=112)	P value
Diagnosis				0.398
IHM	128 (56.4%)	68 (59.1%)	60 (53.6%)	
IMCC	99 (43.6%)	47 (40.9%)	52 (46.4%)	
Sex				0.272
Male	146 (64.3%)	78 (67.8%)	68 (60.7%)	
Female	81 (35.7%)	37 (32.2%)	44 (39.3%)	
Age (years)	66.6 +/- 9.7	67.8 +/- 9.9	65.4 +/- 9.4	0.063
CA19-9 (U/mL)	23.82 (6.22-1731.57)	23.82 (6.19-1907.07)	22.85 (6.35-1259.44)	0.928
CEA (ng/mL)	3.48 (2.57-6.12)	3.49 (2.56-5.95)	3.48 (2.60-6.33)	0.979
AFP (ng/mL)	2.56 (1.83-4.00)	2.56 (1.81-3.88)	2.58 (1.85-4.04)	0.780

### Model performance

**Figure 2:**
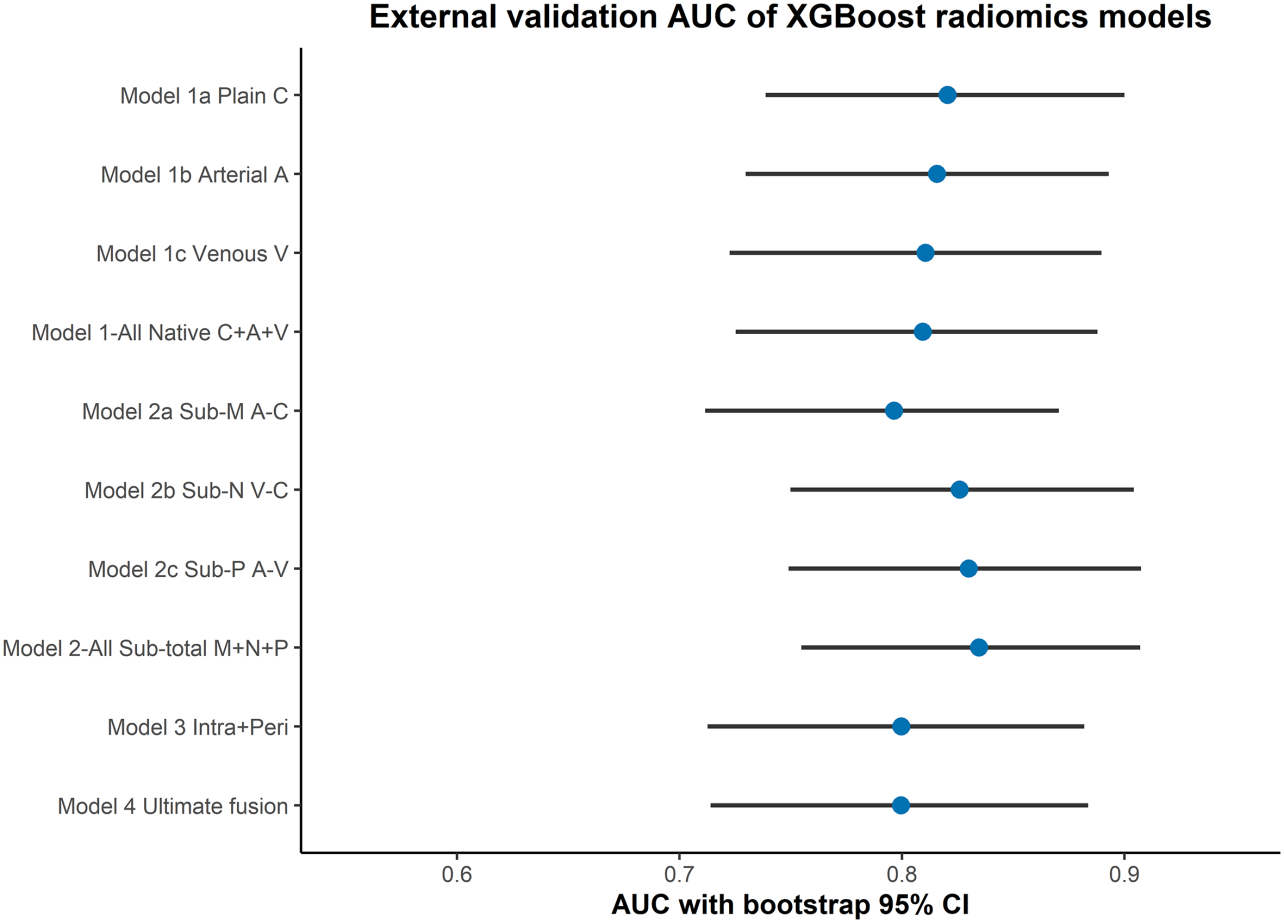
External validation AUC forest plot. Points represent AUCs, and horizontal bars indicate bootstrap 95% confidence intervals for all ten XGBoostmodels.

**Figure 3:**
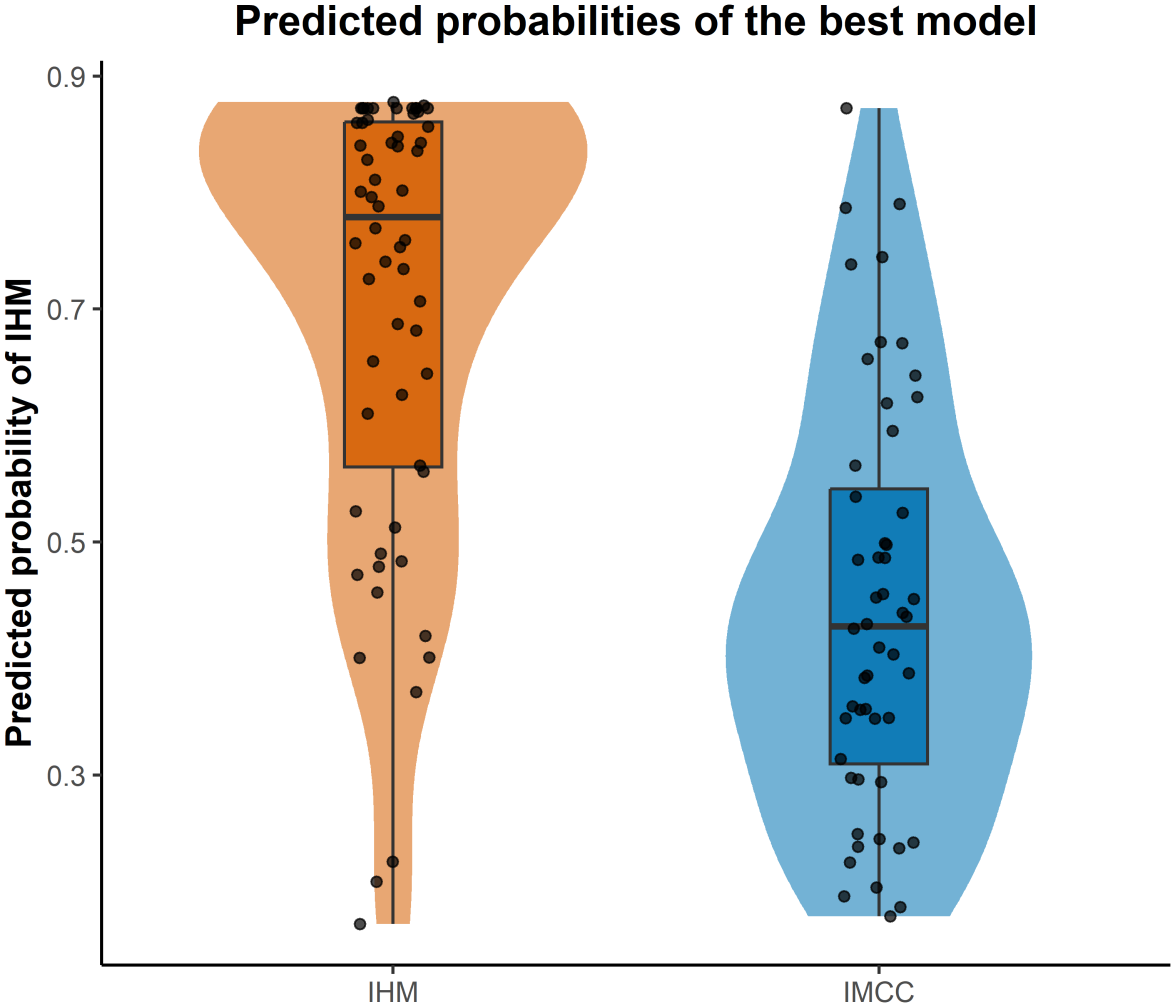
Distribution of predicted probabilities for the best model. The Model 2-All subtraction-total model assigned higher predicted probabilities to IHM than to IMCC in the external validation cohort.

The performance of all ten XGBoost radiomics models is summarized in **Table 2** and **Figure 2**. In the training cohort, five-fold cross-validated AUCs ranged from 0.740 to 0.786, whereas apparent training AUCs ranged from 0.864 to 0.978. The Model 2-All subtraction-total model showed the highest numerical external validation AUC of 0.835 (0.755-0.907), although pairwise DeLong tests did not demonstrate statistically significant differences after multiple-comparison correction. Subtraction-based models ranked among the top-performing models, including Model 2c with an AUC of 0.830 (0.749-0.907) and Model 2b with an AUC of 0.826 (0.750-0.904). In contrast, the native C+A+V model and ultimate fusion model did not numerically outperform the subtraction-total model. The predicted-probability distribution of the best numerical model in the external validation cohort is shown in **Figure 3**.

**Table 2:** XGBoost model performance in training and external validation cohorts.

Model	Feature set	Input features	Selected features	CV AUC	Training AUC	External AUC (95% CI)	External accuracy	Sensitivity	Specificity	PPV	F1 score
Model 1a Plain C	C intratumoral	107	50	0.784	0.902	0.821 (0.739-0.900)	0.777	0.817	0.731	0.778	0.797
Model 1b Arterial A	A intratumoral	107	50	0.780	0.895	0.816 (0.730-0.893)	0.759	0.767	0.750	0.780	0.773
Model 1c Venous V	V intratumoral	107	20	0.786	0.899	0.811 (0.723-0.890)	0.777	0.783	0.769	0.797	0.790
Model 1-All Native C+A+V	C+A+V intratumoral	321	20	0.767	0.864	0.809 (0.725-0.888)	0.759	0.717	0.808	0.811	0.761
Model 2a Sub-M A-C	M=A-C intratumoral	107	5	0.757	0.883	0.796 (0.712-0.871)	0.777	0.717	0.846	0.843	0.775
Model 2b Sub-N V-C	N=V-C intratumoral	107	20	0.754	0.923	0.826 (0.750-0.904)	0.732	0.900	0.538	0.692	0.783
Model 2c Sub-P A-V	P=A-V intratumoral	107	20	0.757	0.881	0.830 (0.749-0.907)	0.777	0.733	0.827	0.830	0.779
Model 2-All Sub-total M+N+P	M+N+P intratumoral	321	50	0.751	0.889	0.835 (0.755-0.907)	0.759	0.800	0.712	0.762	0.780
Model 3 Intra+Peri	C+A+V intra+2-mm peritumoral	321	50	0.775	0.978	0.800 (0.713-0.882)	0.714	0.817	0.596	0.700	0.754
Model 4 Ultimate fusion	C+A+V+M+N+P intra/peritumoral fusion	963	50	0.740	0.867	0.800 (0.714-0.884)	0.759	0.733	0.788	0.800	0.765

Internal and external performance showed different patterns. Several models had high apparent training AUCs, particularly the intra-plus-peritumoral model, but this did not translate into the highest external validation performance. This pattern suggests that external validation is essential for selecting the final model and that apparent training performance alone may overestimate model generalizability.

### Feature selection

**Figure 4:**
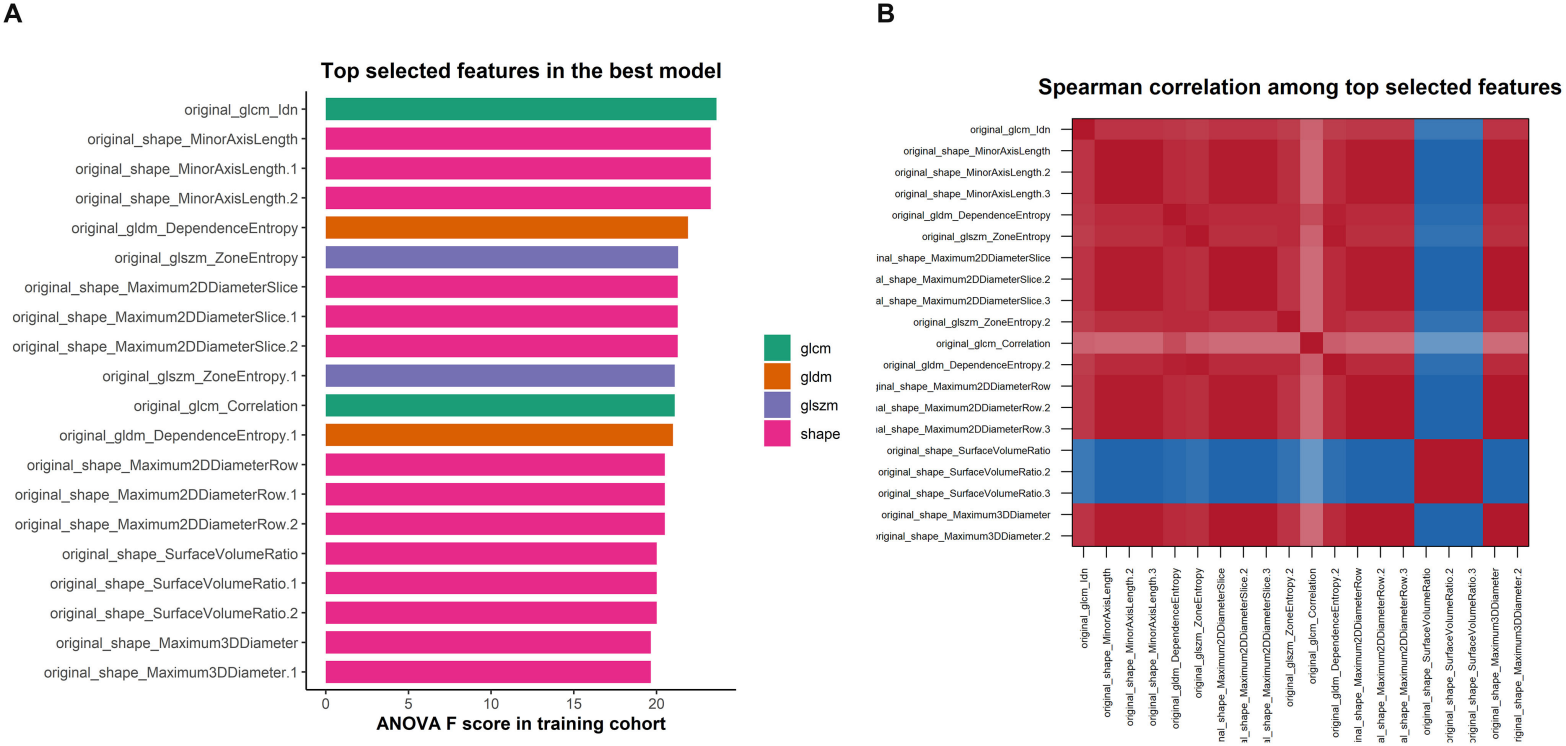
Top selected features and correlation structure of the best model. Panel **(A)** shows the top 20 Model 2-All subtraction-total features ranked by ANOVA F score in the training cohort. Panel **(B)** shows the Spearman correlation matrix of these top-ranked features.

Model 2-All selected 50 features from 321 input subtraction features. These selected features were distributed across all three subtraction maps (M, 15 features; N, 19 features; P, 16 features). By feature family, the selected set included shape features (n=27), gray-level co-occurrence matrix (GLCM) features (n=8), gray-level dependence matrix (GLDM) features (n=5), gray-level size-zone matrix (GLSZM) features (n=5), gray-level run-length matrix (GLRLM) features (n=3), and neighboring gray-tone difference matrix (NGTDM) features (n=2). The top-ranked features and their correlation structure are summarized in **Figure 4**.

### Model comparison, calibration, and clinical utility

**Figure 5:**
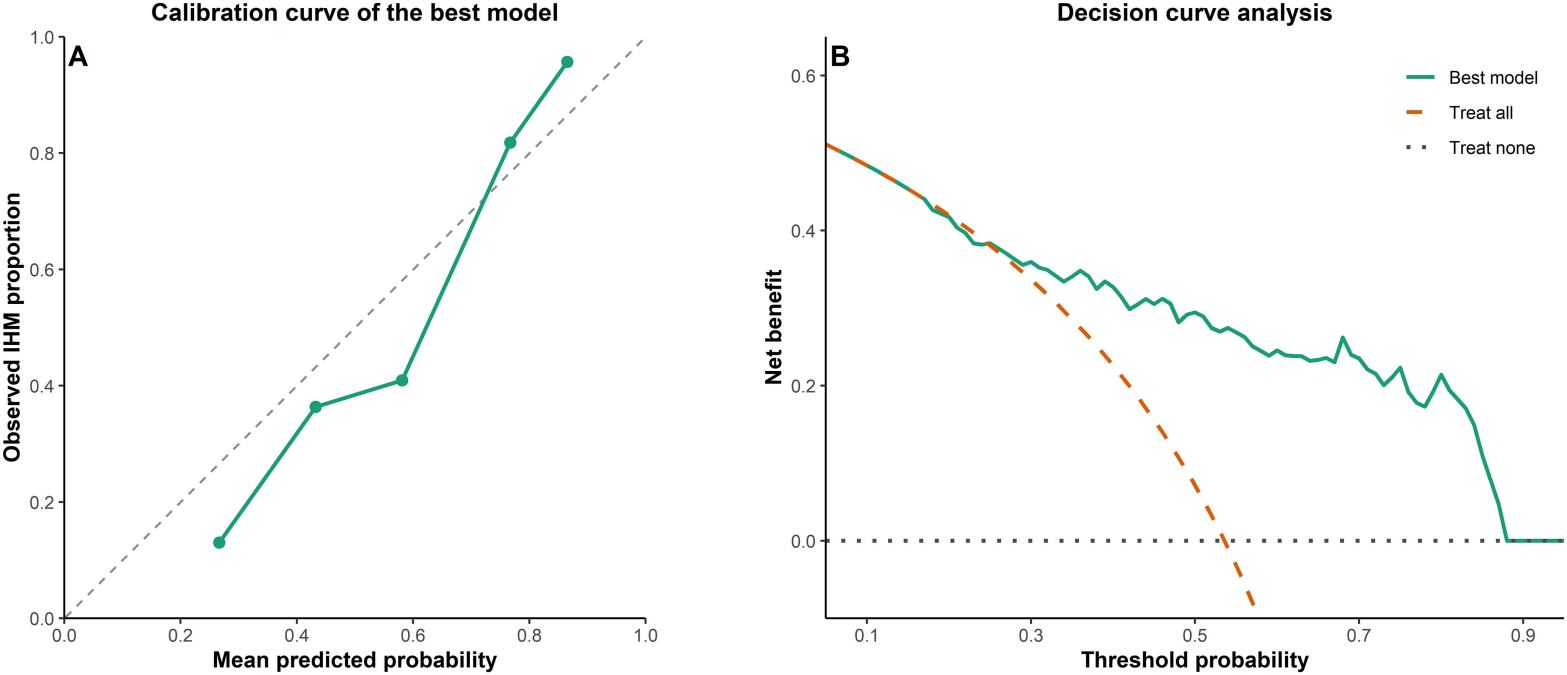
Calibration and decision curve analysis of the best model. Panel **(A)** shows the calibration curve of the Model 2-All subtraction-total model in the external validation cohort. Panel **(B)** shows decision curve analysis comparing the best model with treat-all and treat-none strategies.

Pairwise DeLong tests among XGBoost models did not demonstrate statistically significant differences after multiple-comparison correction. The best subtraction-total model showed a generally monotonic calibration pattern in the external validation cohort, although some deviation remained, and decision curve analysis indicated potential net benefit across a range of clinically plausible threshold probabilities (**Figure 5**).

## Discussion

This two-center study developed and externally validated ten multiphase CT radiomics models for differentiating IHM from IMCC. Using center 1 for training and center 2 for external validation, the subtraction-total XGBoost model based on M+N+P intratumoral features achieved the highest numerical performance, with an external validation AUC of 0.835 (0.755-0.907). However, this difference was not statistically significant after multiple-comparison correction, and the result should therefore be interpreted as numerical rather than definitive superiority. These findings suggest that subtraction-derived enhancement-difference information may provide useful discriminatory information beyond native CT phases.

The performance pattern is clinically plausible and is consistent with recent interest in multiphase and enhancement-aware CT radiomics for liver tumors. A recently published multiphase CT dataset for automated differential diagnosis of liver tumors highlights the clinical value of using noncontrast, arterial, venous, and delayed-phase information together rather than relying on a single static image (5). In intrahepatic cholangiocarcinoma, recent contrast-enhanced CT radiomics studies have shown that quantitative features from tumor habitats, enhancement patterns, and whole-lesion phenotypes can provide information related to lymph node metastasis, prognosis, and clinical outcome (18–20). These studies do not directly address IHM versus IMCC classification, but they support the broader premise that dynamic enhancement information in CT contains diagnostically relevant tumor phenotypes.

From a pathophysiologic perspective, the subtraction maps used here represent different aspects of enhancement kinetics. M=A-C emphasizes net arterial enhancement, N=V-C emphasizes net venous enhancement, and P=A-V reflects the difference between arterial and venous enhancement, which may relate to washout or persistent enhancement. IHM and IMCC may differ in vascular supply, fibrous stroma, necrosis, tumor cellularity, and delayed enhancement. Native CT features mainly encode morphology and baseline attenuation heterogeneity, whereas subtraction features encode spatial heterogeneity in enhancement change. Recent CT delta-radiomics and spatiotemporal radiomics studies have similarly used quantitative feature changes to characterize tumor behavior beyond a single baseline image (21, 22). Although those studies focused on other tumor types and longitudinal treatment settings, they provide a contemporary methodological analogue for the present phase-difference radiomics strategy. This distinction may explain why Model 2b, Model 2c, and Model 2-All ranked highly in external validation.

The selected features of the best numerical model provide a useful, although exploratory, explanation for this result. Model 2-All selected 50 features from the three subtraction maps, with a balanced contribution from M, N, and P. Shape features accounted for 27 selected features, including axis length, maximum diameter, sphericity, and surface-to-volume ratio. Because the same intratumoral ROI was applied across subtraction sequences, these shape features should be interpreted primarily as lesion size and compactness anchors rather than as subtraction-specific morphology. More mechanistically informative were the selected texture features. GLCM features such as joint entropy, inverse difference normalized, inverse difference moment normalized, informational measure of correlation, and correlation describe spatial organization and disorder of gray-level relationships. GLDM, GLSZM, GLRLM, and NGTDM features, including dependence entropy, small-dependence emphasis, zone entropy, zone percentage, run-length nonuniformity normalized, short-run emphasis, and coarseness, quantify local dependence, zone size, run patterns, and neighborhood gray-tone variation. In subtraction images, these descriptors can be read as quantitative summaries of enhancement heterogeneity rather than raw attenuation heterogeneity. This interpretation is consistent with standardized radiomics feature definitions and recent contrast-enhanced CT radiomics studies in liver malignancies (16, 18–20).

This feature pattern also links the radiomics model to the biologic contrast between the two diseases. A lesion with irregular arterial enhancement, heterogeneous venous retention, necrotic components, or fibrotic delayed enhancement may generate higher entropy, fragmented zones, shorter runs, or altered local dependence on M, N, and P maps. Conversely, more homogeneous enhancement behavior may produce smoother texture organization. These associations remain hypothesis-generating, but they make the subtraction-total model clinically interpretable: it does not simply add more images, but summarizes how enhancement changes are spatially distributed inside the lesion.

The ultimate fusion model did not achieve the highest external validation AUC. This result is important because it indicates that adding more feature sources does not necessarily improve generalization. Fusion models may increase dimensionality, redundancy, and center-specific noise, particularly in moderate-sized datasets. Similarly, the intra-plus-peritumoral model did not outperform the best subtraction models, suggesting that the 2-mm peritumoral region may not add stable incremental information under the current segmentation and modeling settings. This does not negate the potential value of peritumoral radiomics, but it suggests that its contribution may depend on tumor type, margin biology, segmentation reproducibility, and sample size.

Several limitations should be acknowledged. First, although baseline clinical variables and tumor markers were summarized, this study did not construct clinical-only or combined clinical-radiomics models. Second, although the CT acquisition protocol was described and standardized preprocessing was applied, scanner heterogeneity between centers may still have affected radiomics feature distributions. ComBat harmonization was considered but was not applied in the current analysis because of the moderate sample size and the center-based external validation design; future larger multicenter studies should evaluate whether harmonization improves model transportability. Third, although subtraction models showed the highest numerical performance, pairwise statistical comparisons did not establish robust superiority after correction for multiple testing. Fourth, the final reported feature sets were selected after refitting on the full training cohort, and formal fold-wise feature-stability analysis was not performed. Fifth, feature interpretation is associative and should not be treated as direct histopathologic proof of vascularity, fibrosis, necrosis, or stromal composition. Sixth, the study sample size remains moderate and additional multicenter validation is required before clinical implementation.

## Conclusion

SelectKBest-based XGBoost radiomics models derived from multiphasic contrast-enhanced CT showed promising performance for differentiating IHM from IMCC in a two-center setting. Subtraction-image-based intratumoral models, especially the M+N+P subtraction-total model, achieved the highest numerical external validation performance, although this finding did not indicate statistically significant superiority after multiple-comparison correction. These findings support the potential value of enhancement-difference radiomics features, while also emphasizing the need for larger multicenter validation and integration with clinical variables.

## Data Availability

The raw data supporting the conclusions of this article will be made available by the authors, without undue reservation.
