# Supplementary material for: Comparative multiphase CT radiomics for differentiating isolated hepatic metastasis from intrahepatic cholangiocarcinoma: a two-center study of native, subtraction, peritumoral, and fusion models
Source: Front Oncol. 2026 Jul 30;16:1907000. doi: 10.3389/fonc.2026.1907000 (PMC13467766; doi:10.3389/fonc.2026.1907000)
Supplement: Supplementary file 1 [file Table1.docx]

**Supplementary Table S1. Feature Selection and XGBoost Hyperparameters**

All feature-selection and model-tuning steps were performed only in the training cohort. Candidate SelectKBest values were 5, 10, 20, 50, and all features when applicable.

| **Model** | **Input features** | **After variance filter** | **Selected features** | **Selection k** | **max_depth** | **n_estimators** | **learning_rate** |
| --- | --- | --- | --- | --- | --- | --- | --- |
| Model 1a Plain C | 107 | 107 | 50 | 50 | 1 | 80 | 0.1 |
| Model 1b Arterial A | 107 | 107 | 50 | 50 | 1 | 150 | 0.05 |
| Model 1c Venous V | 107 | 107 | 20 | 20 | 1 | 80 | 0.05 |
| Model 1-All Native C+A+V | 321 | 321 | 20 | 20 | 1 | 80 | 0.05 |
| Model 2a Sub-M A-C | 107 | 107 | 5 | 5 | 1 | 80 | 0.1 |
| Model 2b Sub-N V-C | 107 | 107 | 20 | 20 | 1 | 150 | 0.1 |
| Model 2c Sub-P A-V | 107 | 107 | 20 | 20 | 1 | 150 | 0.05 |
| Model 2-All Sub-total M+N+P | 321 | 321 | 50 | 50 | 1 | 150 | 0.05 |
| Model 3 Intra+Peri | 321 | 321 | 50 | 50 | 1 | 150 | 0.05 |
| Model 4 Ultimate fusion | 963 | 963 | 50 | 50 | 1 | 80 | 0.05 |
